# TB Vaccines: The (Human) Challenge Ahead

**DOI:** 10.4172/2161-1068.1000e128

**Published:** 2014-02-25

**Authors:** David A Hokey

**Affiliations:** Department of Immunology, Aeras, Rockville, Maryland, USA

Tuberculosis (TB) remains a global health threat, with 8.6 million new cases and 1.3 million deaths (including 0.3 million in HIV-positive people) in 2012 [1]. An estimated one-third of the world's population is infected with *Mycobacterium tuberculosis* (Mtb) [2]. TB is one of the leading causes of death by an infectious disease worldwide, second only to HIV (1.6 million deaths in 2012), and the increase in drug resistant strains of Mtb is alarming [1,3]. An effective vaccine to prevent infection and/or pulmonary TB disease is desperately needed. However, there are several hurdles that have made TB vaccine research and development painfully slow, including the slow growth rate of Mtb, the lack of a predictive animal model, and the lack of an immune correlate [3]. In addition, animal challenge models are very expensive due to the requirement of an ABSL-3 facility, and clinical efficacy studies are long and require large numbers of patients. Funding continues to be an issue in the TB vaccine field (Figure 1) [1,4-6], particularly given the difficulties and the expense of preclinical and clinical studies. New tools are needed to evaluate candidate vaccines that produce a more rapid and accurate assessment of the potential for vaccine efficacy and, therefore, help to increase the speed, and reduce the cost, of vaccine candidate selection.

Given the absence of an immune correlate, functional assays that can assess the ability of a vaccine to inhibit mycobacterial growth are needed to give some measure of assurance of the efficacy of a vaccine candidate. There has been some progress in this area, such as the mycobacterial growth inhibition assay (MGIA), which is currently being developed by a consortium of international scientists and organizations. The MGIA has the potential to be a surrogate for protection and may lead to the identification of immune correlates [7,8]. However, it remains to be seen whether this *in vitro* assay correlates with any type of protection in humans.

Malaria vaccine development has experienced similar challenges to the TB vaccine field given the lack of an immune correlate [9,10]. While smaller clinical trials can assess the safety and immunogenicity of a vaccine, large and expensive efficacy studies generally are needed to assess whether the observed immune responses are able to impact disease. A human challenge model of malaria infection has helped malaria vaccine developers to overcome these obstacles and, in so doing, has transformed the malaria vaccine field, providing a relatively inexpensive and rapid method for assessing vaccine efficacy prior to the initiation of very expensive phase IIb and phase III clinical efficacy trials [10]. The malaria human challenge model involves the vaccination of healthy volunteers who are then subjected to experimental malarial infection by mosquito bite. The volunteers are followed until parasites are detected in the blood, at which point they are treated with anti-malarial medication [10,11]. The malaria human challenge model has been shown to be safe with over 1,450 volunteers challenged by this method [10-12]. Results from the RTS,S trial appear to confirm the predictive value of the human challenge model [10].

The development of a human challenge model for tuberculosis is not as straight forward as for malaria. While there are ethical concerns for any human challenge model [13], exposing healthy individuals to virulent Mtb poses significant safety concerns and is ethically unacceptable. One possible alternative is to use the licensed vaccine for TB, Bacille Calmette-Guérin (BCG), as a surrogate for Mtb. BCG is a replication-competent, live attenuated mycobacterium derived from *Mycobacterium bovis* that offers limited and variable protection against disseminated forms of childhood TB. BCG is administered intradermally as a live vaccine that is cleared in immunocompetent vaccinees, eliciting similar immune responses to those observed following Mtb infection [14]. Because of its long safety record in healthy infants and intradermal administration, BCG is attractive for use for human challenge studies in healthy adults.

Helen McShane's group at Oxford University developed a preclinical *in vivo* murine challenge model using BCG. In this model, they challenged mice previously administered a TB vaccine with an intradermal injection of BCG in the ear and measured the change in BCG burden over time. The authors demonstrated that the intradermal BCG challenge in the ear mimicked an intranasal challenge, suggesting this model is relevant for assessment of protection [14]. This work provided the basis for the development of a human challenge model. In 2012, Minassian et al. published data describing a human challenge study with BCG. The researchers challenged both BCG-naïve and BCG-vaccinated volunteers with intradermal BCG injections and subsequently collected biopsies and performed PCR and cultures to measure the number of bacteria present at the injection site. In addition, they collected fluid from suction cup blisters to examine cellular infiltrates and measured immune responses in the peripheral blood. The authors observed differences in the bacterial counts obtained from different volunteers that had been previously vaccinated with BCG, which they hypothesized could be due to varying levels of exposure to environmental mycobacteria. There were some concerns regarding the study. First, the PCR and culture counts were not in agreement, which could have been due to either PCR counting of dead bacteria or possibly due to differences in the time when samples were plated. In addition, because volunteers were enrolled over the course of two years, different batches of BCG were needed for vaccination, with batches having a variability of approximate 1-log of bacteria [15]. The results of this study suggest that human challenge with BCG is safe and feasible, although in need of further optimization.

While the potential for a BCG challenge model is encouraging, there are a few issues that must be addressed. The challenge strain should be capable of evaluating all, or most, of the vaccine candidates that are under development. While BCG has much in common with Mtb, there are differences, and vaccines that target antigens unique to Mtb would not be suitable for the BCG challenge model. Potential ways to circumvent this problem would be to use either a recombinant BCG that expresses the unique Mtb antigens or to use attenuated Mtb, which is currently being investigated as a vaccine candidate [16]. Ideally, the challenge model would be less invasive and not require the use of biopsies to acquire samples from volunteers. Prior studies examined swabbing the infection area in order to culture any shed bacteria from the surface [17]. It may also be possibly to utilize luminescence or other novel non-invasive methods to quantify bacteria at the challenge site. Additionally, the challenge dose must be standardized. If the dose range varies by a log or more, the ability to distinguish small protective responses is greatly diminished. Such differences ultimately may not impact the readout of the assay, but more work needs to be performed to specifically address this issue.

A study has been submitted to ClinicalTrials.gov to examine dose escalation for the human BCG challenge model, with challenge doses ranging from 2-16×10^6^ CFU, in order to assess safety, shedding, and reproducibility of shedding. The researchers will utilize PCR, culture, and quantitative MGIT BACTEC culture to assess bacterial shedding [18]. This study has not started as of this writing but the results may have a significant impact on the future of this model and may help to address some of the concerns regarding the variability of the challenge stock.

The human challenge model could change the field of TB vaccine development as the malaria human challenge model did for malaria vaccines, not only by providing a less expensive and rapid method for assessing potential vaccine efficacy, but also by permitting more rapid progress toward the identification of an immune correlate of protection. While the challenges in developing this model are great, it is clear that the research community is embracing this challenge.

## Figures and Tables

**Figure 1:**
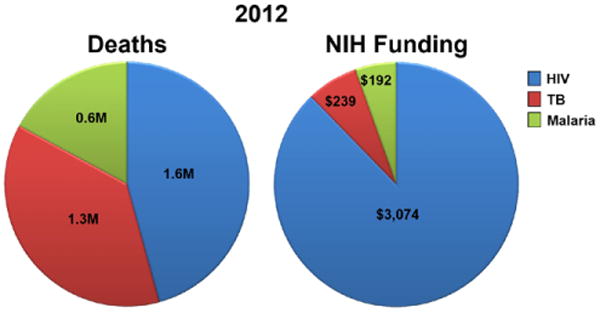
NIH funding for HIV, TB, and malaria Pie charts represent the approximate proportion of deaths (in millions; left pie) and the level of NIH funding (in millions; right pie) for HIV (blue), TB (red), and malaria (green). Data is based on the Estimates of Funding for Various RCDC, Global Tuberculosis Report 2013, World Malaria Report 2013, and the Global Report: UNAIDS Report on the Global AIDS Epidemic 2013.
